# Near-atomic resolution reconstructions from *in situ* revitrified cryo samples

**DOI:** 10.1107/S2059798323003431

**Published:** 2023-05-23

**Authors:** Gabriele Bongiovanni, Oliver F. Harder, Jonathan M. Voss, Marcel Drabbels, Ulrich J. Lorenz

**Affiliations:** a Laboratory of Molecular Nanodynamics, Ecole Polytechnique Federale de Lausanne (EPFL), CH-1015 Lausanne, Switzerland; University of Glasgow, United Kingdom

**Keywords:** microsecond melting and revitrification, microsecond time-resolved cryo-EM, protein dynamics, time-resolved electron microscopy, preferential orientation

## Abstract

A microsecond time-resolved version of cryo-electron microscopy (cryo-EM) has recently been introduced to enable observation of the fast conformational motions of proteins. The technique involves locally melting a cryo sample with a laser beam to allow the proteins to undergo dynamics in the liquid phase. When the laser is switched off, the sample cools within just a few microseconds and revitrifies, trapping particles in their transient configurations, in which they can subsequently be imaged. Two alternative implementations of the technique have previously been described, using either an optical microscope or performing revitrification experiments *in situ*. Here, it is shown that it is possible to obtain near-atomic resolution reconstructions from *in situ* revitrified cryo samples. Moreover, the resulting map is indistinguishable from that obtained from a conventional sample within the spatial resolution. Interestingly, it is observed that revitrification leads to a more homogeneous angular distribution of the particles, suggesting that revitrification may potentially be used to overcome issues of preferred particle orientation.

## Abstract 2

Near-atomic resolution reconstructions can be obtained from *in situ* melted and revitrified cryo samples. Revitrification results in a more homogeneous angular distribution.

## 1. Introduction

Proteins play a crucial role in most biological processes. They catalyze reactions, regulate gene expression, receive and transmit biological signals, and participate in the recognition of pathogens (Benkovic & Hammes-Schiffer, 2003 ▸; Buccitelli & Selbach, 2020 ▸; Wootten *et al.*, 2018 ▸; Kumar *et al.*, 2009 ▸). To perform their tasks, they undergo large-scale domain motions that bear resemblance to those of manmade machines (Alberts, 1998 ▸). However, because of the fast timescale of these motions, typically microseconds, it has largely remained impossible to observe them directly, which has left our understanding of protein function fundamentally incomplete (Henzler-Wildman & Kern, 2007 ▸; Otosu *et al.*, 2015 ▸; Persson & Halle, 2008 ▸). In fact, it has been argued that understanding protein function represents the next frontier in structural biology (Ourmazd *et al.*, 2022 ▸).

We have recently introduced a novel approach to time-resolved cryo-EM that affords microsecond time resolution. It thus promises to significantly advance our understanding of protein function by enabling direct observations of the motions of proteins (Voss *et al.*, 2021*a* ▸,*b* ▸; Harder *et al.*, 2022 ▸). Our approach involves rapidly melting a cryo sample with a laser beam. Once the sample is liquid, conformational dynamics are triggered with a suitable stimulus, for example by releasing a caged compound or directly exciting a photoactive protein with a second laser pulse (Klein-Seetharaman, 2002 ▸; Ellis-Davies, 2007 ▸; Shigeri *et al.*, 2001 ▸). As the particle dynamics unfold, the heating laser is switched off at a well defined point in time and the sample revitrifies within just a few microseconds, trapping the particles in their transient configurations, in which they can subsequently be imaged with conventional cryo-EM techniques (Voss *et al.*, 2021*b* ▸).

We have previously described two different implementations of our technique. Melting and revitrification experiments can be performed in an optical microscope using a correlative light-electron microscopy approach (Bongiovanni *et al.*, 2022 ▸). Alternatively, such experiments can be carried out *in situ* (Voss *et al.*, 2021*a* ▸,*b* ▸; Harder *et al.*, 2022 ▸) with a modified transmission electron microscope (Olshin *et al.*, 2020 ▸). *In situ* experiments offer the advantage that electron micrographs of revitrified areas can provide immediate feedback on conformational changes of the proteins. It is even possible to perform on-the-fly reconstructions to guide the search for suitable experimental parameters. Moreover, it is straightforward to determine the diameter of the revitrified area from electron micrographs, which can be monitored to adjust the laser power in revitrification experiments, as we have previously described (Voss *et al.*, 2021*a* ▸). A potential drawback of *in situ* experiments is that the sample has to be exposed to a small electron dose (about 10^−3^ e Å^−2^) in order to locate a suitable area and aim the laser beam. This is a potential issue since we have previously observed that exposure to a dose as low as a few e Å^−2^ prior to revitrification induces enough beam damage to cause the particles to disassemble once the sample has become liquid (Voss *et al.*, 2021*a* ▸,*b* ▸). We have recently demonstrated that *in situ* revitrification does not alter the structure of the proteins (Harder *et al.*, 2022 ▸). However, the resolution of the reconstructions we obtained was limited by the performance of our instrument to a few angstroms. This leaves open the question whether *in situ* revitrification might induce structural changes that only become evident at higher spatial resolution or might even limit the obtainable resolution.

Here, we show that near-atomic resolution reconstructions can be obtained from *in situ* revitrified cryo samples by transferring them to a high-resolution electron microscope for cryo imaging. Interestingly, our analysis reveals that rapid melting and revitrification reshuffles the particles, creating a more homogeneous angular distribution.

## 2. Methods

Cryo specimens were prepared on UltrAuFoil R1.2/1.3 300 mesh grids (Quantifoil). The grids were plasma cleaned for 1 min to render them hydrophilic (Ted Pella ‘EasyGlow’). Subsequently, 3 µl of the sample solution (mouse heavy chain apoferritin, 8.5 mg ml^−1^ in 20 m*M* HEPES buffer with 300 m*M* sodium chloride at pH 7.5) was applied and the samples were plunge-frozen using a Vitrobot Mark IV (Thermo Fisher Scientific, 3 s blotting time, 95% relative humidity at a temperature of 10°C).

*In situ* revitrification experiments were carried out with a modified Jeol 2200FS transmission electron microscope (Olshin *et al.*, 2020 ▸) using a Gatan Elsa single-tilt cryo holder. Microsecond laser pulses for melting and revitrification (76 mW) were obtained by modulating the output of a 532 nm continuous-wave laser (Laser Quantum, Ventus 532) with an acousto-optic modulator (AA Optoelectronics). The beam was focused in the sample plane, giving an elliptical spot of 62 × 165 µm FWHM, as determined from a knife-edge scan. Before transferring the sample for high-resolution imaging an atlas was recorded, which allowed us to readily identify the revitrified areas later.

The micrographs in Fig. 3(*b*) were recorded with the Jeol 2200FS. High-resolution micrographs for single-particle reconstructions (Figs. 2 and 3) were collected on a Titan Krios G4 (Thermo Fisher Scientific), which was operated at an acceleration voltage of 300 kV. Zero-loss filtered images (Selectris X energy filter, 10 eV slit width) were recorded with a Falcon 4 camera, using an exposure time of about 2.5 s for a total dose of 50 e Å^−2^. The pixel size was set to 0.455 Å. Defocus values were in the range 0.5–1.2 µm.

Single-particle reconstructions were performed in *cryo­SPARC* 3.3.1 (Punjani *et al.*, 2017 ▸; details are given in the supporting information). The conventional and revitrified data sets, both of which were recorded on the same specimen grid, contained 4745 and 1591 images, respectively, which were patch motion-corrected. After CTF estimation, 1815 and 1552 micrographs with a resolution better than 6 Å were retained for the conventional and revitrified data sets, respectively. Template-based particle picking yielded 114 414 and 88 004 particles, respectively. After two rounds of 2D classification, 91 541 and 74 468 particles were retained for the conventional and revitrified data sets, respectively, which were used for *ab initio* reconstruction (*C*1 symmetry), followed by heterogeneous refinement using two classes (*C*1 symmetry). The 72 270 and 72 811 particles in the most populated class for the conventional and revitrified data sets, respectively, were then used for homogeneous refinement with *O* symmetry, giving maps with 1.61 and 1.59 Å resolution, respectively.

## 3. Results and discussion

**Figure 1:**
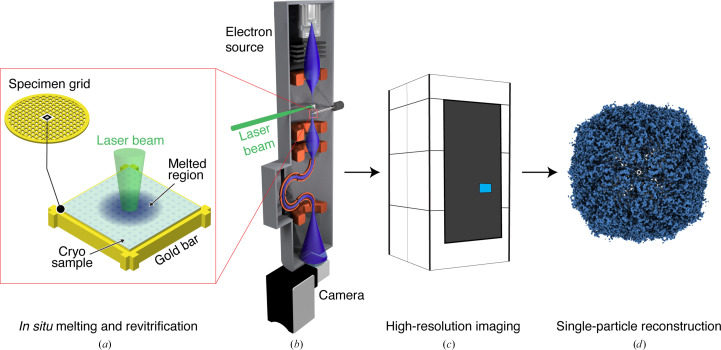
Illustration of the workflow used to obtain a near-atomic resolution reconstruction from *in situ* revitrified cryo samples. (*a*, *b*) Cryo samples on UltrAuFoil grids are melted and revitrified *in situ* with a modified Jeol 2200F transmission electron microscope. (*c*) The samples are then transferred to a Titan Krios G4 for high-resolution imaging. (*d*) Single-particle reconstructions from the revitrified areas yield near-atomic resolution maps.

Fig. 1 ▸ illustrates the experimental workflow. A cryo sample of mouse heavy-chain apoferritin was prepared on a UltrAuFoil specimen support, which features a holey gold film (1.2 µm diameter holes) on a 300 mesh gold grid (Fig. 1 ▸ *a*). *In situ* melting and revitrification experiments were then performed with a Jeol 2200FS transmission electron microscope that we have modified for time-resolved experiments (Fig. 1 ▸ *b*; Olshin *et al.*, 2020 ▸). The melting laser (532 nm) is directed at the sample with the help of an aluminium mirror placed above the upper pole piece of the objective lens, so that the laser beam strikes the sample at close to normal incidence. To perform a melting and revitrification experiment, the laser beam was aimed at the center of a grid square and a 20 µs laser pulse was used to revitrify an area of typically about 25 holes. The sample was then transferred to a Titan Krios G4 (Thermo Fisher Scientitic) for high-resolution imaging (Fig. 1 ▸ *c*). Micrographs were collected from 16 revitrified areas as well as from 11 conventional areas that had not been exposed to the laser beam. Finally, single-particle reconstructions of both data sets were performed with *cryoSPARC* (Punjani *et al.*, 2017 ▸) to yield near-atomic resolution maps (Fig. 1 ▸ *d*).

**Figure 2:**
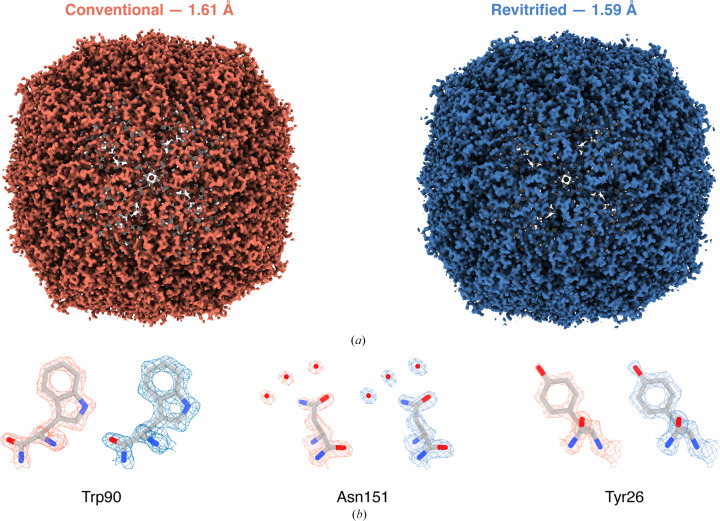
Near-atomic resolution reconstructions of apoferritin from conventional and revitrified areas. (*a*) The maps from conventional (left) and revitrified (right) areas are indistinguishable within the resolution obtained (∼1.6 Å). (*b*) Details of the reconstructions show well resolved side-chain densities as well as individual water molecules. A model of apoferritin (Wu *et al.*, 2020 ▸) is placed into the density by rigid-body fitting.

Fig. 2 ▸ compares the reconstructions obtained from the conventional areas (left) and revitrified areas (right). Within our resolution (1.61 and 1.59 Å, respectively), the maps are undistinguishable. This is also evident in the details of side chains shown in Fig. 2 ▸(*b*), which reveal well resolved densities of aromatic rings as well as individual water molecules. A model of mouse heavy-chain apoferritin is shown (Wu *et al.*, 2020 ▸) that we have placed into the density by rigid-body fitting. These results confirm our previous observations that the *in situ* melting and revitrification process leaves the proteins intact (Harder *et al.*, 2022 ▸). Moreover, we can determine to atomic scale precision that under our experimental conditions *in situ* revitrification does not alter the structure of the particles, nor does it reduce the obtainable spatial resolution. Evidently, the small electron dose of about 10^−3^ e Å^−2^ that the sample is exposed to in an *in situ* experiment prior to revitrification does not cause sufficient beam damage to deteriorate the resolution.

**Figure 3:**
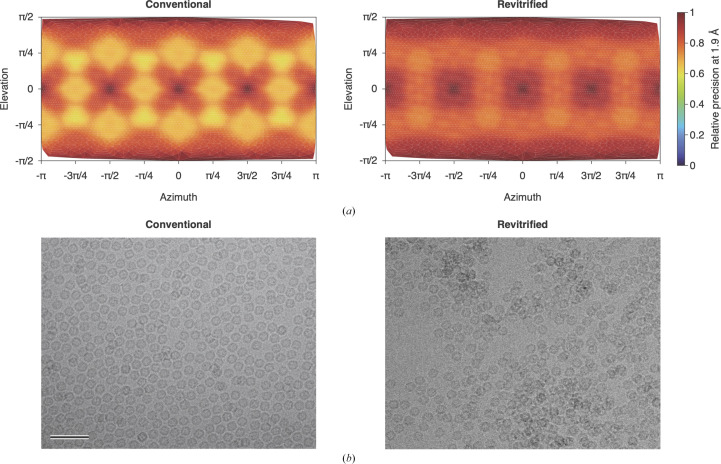
Revitrification reshuffles the particles, which results in a more homogeneous angular distribution. (*a*) Relative precision of the reconstructions at 1.9 Å as a function of angle in the conventional (left) and revitrified (right) sample areas with octahedral symmetry applied. The relative precision reported by *cryoSPARC* approximately reflects the number of observations contributing information to a specific angle. (*b*) A micrograph of a conventional sample area shows a homogeneous distribution of the particles (left), whereas an uneven particle distribution is observed in revitrified areas (right).

Interestingly, melting and revitrification results in a more homogeneous angular distribution of the particles. This is evident in Fig. 3 ▸(*a*), which compares the precision of the reconstructions (at 1.9 Å) as a function of angle for the conventional (left) and revitrified (right) sample areas. The variation in precision results from a preference of the particles for specific orientations, which therefore contribute more information to the corresponding angles (see also the angular distributions in Supplementary Fig. S2). Preferred particle orientations in cryo samples are thought to arise due to the adhesion of hydrophobic regions of the protein surface to the air–water interface (Taylor & Glaeser, 2008 ▸). Such effects are small for apoferritin, which exhibits little orientational preference. The precision of the reconstructions shown in Fig. 3 ▸(*a*) features six narrow, symmetry-equivalent maxima. Each is surrounded by eight broad, shallow minima, which fall into two groups of four that are related to each other by symmetry. After revitrification, this distribution is markedly broadened, with the ratio of the highest to the lowest precision decreasing from 1.6 to 1.3.

The broadening of the angular distribution upon revitrification is accompanied by changes in the spatial distribution of the particles, which provides hints as to the underlying mechanism. Fig. 3 ▸(*b*) shows that while conventional samples typically feature a homogeneous particle distribution (left), revitrification results in an uneven distribution, with the particles clustering in some areas. Evidently, rapid laser melting of the sample reshuffles the particles and creates a non-equilibrium spatial and angular distribution. The time window during which the sample is liquid (<20 µs) is manifestly insufficient to re-establish an equilibrium distribution as is initially established during the plunge-freezing process, where the sample is given about 1 s to equilibrate between blotting and plunging. Revitrification therefore traps a non-equilibrium distribution. This is also consistent with the expected rotational timescales. The r.m.s. rotation angle of freely diffusing apoferritin is about 80° on a timescale of 10 µs. However, particles near a surface frequently experience so-called anomalous diffusion, with diffusion coefficients lowered by several orders of magnitude (Jamali *et al.*, 2021 ▸). The r.m.s. rotation angle in our experiment is therefore likely to be lower than the separation between the maxima and minima of the angular distribution (Fig. 3 ▸ *a*).

Several phenomena may potentially explain the reshuffling of the particles during the melting process. As we have previously described, the sample partially crystallizes during rapid laser heating when it traverses so-called ‘no man’s land’ (Voss *et al.*, 2021*a* ▸). It is conceivable that the formation of ice crystallites and their subsequent melting exert small forces upon the particles that scramble their distribution. Another possibility is that stresses present in the vitreous ice film that build up during plunge-freezing due to the difference in expansion coefficient between gold and water (Thorne, 2020 ▸) are unevenly released during rapid laser melting, causing a microscopic flow of the liquid to reshuffle the particles. Further experiments will be needed to definitively settle this point.

## 4. Conclusion

Our experiments demonstrate that near-atomic resolution reconstructions can be obtained from *in situ* melted and revitrified cryo samples, in agreement with previous results for samples that we revitrified in an optical microscope (Bongiovanni *et al.*, 2022 ▸). This strongly suggests that the revitrification process does not impose a fundamental limit on the obtainable spatial resolution. Evidently, the small electron dose that the sample is exposed to in an *in situ* experiment prior to melting and revitrification does not alter the protein structure or the achievable resolution. This is an important result since *in situ* experiments offer the advantage over revitrification with an optical microscope that they can provide immediate feedback, which speeds up the search for suitable experimental parameters. As our technique is applied to a wider range of systems, the circumstances under which this advantage can outweigh the greater simplicity of revitrification experiments in an optical microscope will become clear. If *in situ* revitrification experiments can be directly performed in a modified high-resolution cryo microscope, this would certainly facilitate the workflow by eliminating a sample-transfer step. Such an approach may be particularly interesting for user facilities.

The observed scrambling of the angular distribution of the particles during revitrification may potentially offer a means to overcome issues with preferred orientation that cause views to be underrepresented or even absent in some samples, making it difficult to obtain a reconstruction (Tan *et al.*, 2017 ▸). While tilting the sample during imaging can be used to mitigate this effect (Penczek & Frank, 2006 ▸; Tan *et al.*, 2017 ▸), tilting to high angles reduces the obtainable resolution (Tan *et al.*, 2017 ▸). Instead, laser revitrification may potentially be employed to repopulate hidden views without the need for tilting. Further experiments are currently under way in our laboratory to find experimental parameters that maximize the effect and to determine whether reorientation can also be achieved in samples of highly asymmetric proteins that exhibit more prononounced issues with preferred orientation than apoferritin, where the high symmetry of the particles allows a high-resolution reconstruction despite an uneven angular distribution.

## 5. Data-availability statement

The data that support the findings of this study are available from the corresponding author upon reasonable request. The maps of apoferritin obtained from conventional and revitrified sample areas have been deposited in the EMDB (EMD-15715 and EMD-15721, respectively) and the corresponding data sets have been deposited in EMPIAR (EMPIAR-11345 and EMPIAR-11460, respectively).

## Supplementary Material

EMDB reference: apoferritin, conventional sample, EMD-15715

EMDB reference: revitrified sample, EMD-15721

Details of the single-particle reconstructions and Supplementary Figures. DOI: 10.1107/S2059798323003431/id5012sup1.pdf
